# A survey of Irish psychiatric trainees attitudes to balint groups

**DOI:** 10.1192/bjo.2021.691

**Published:** 2021-06-18

**Authors:** Sudha Jain, Emma Adams, Alyson Lee

**Affiliations:** 1 Mid East Kildare West/ Wicklow Menatal Health Services; 2 Kildare West Wicklow Mental Healt Services

## Abstract

**Aims:**

1. To compare the experience of psychiatric trainees in Ireland of online Balint Groups (BG) in contrast to face to face groups.

2. To assess the general attitudes of trainees to BG using the Psychological Medical Inventory (PMI) (Ireton and Sherman, 1988) scale.

**Method:**

An online survey comprising two sections: 1. A questionnaire exploring participants experience, effectiveness and obstacles to attending the two formats of BG with a free text box response. 2. General attitude of trainees towards BG using PMI scale.

An online cross-sectional survey using Survey Monkey. An invitation to participate in the survey was emailed to all trainees by the College of Psychiatrists in Ireland. All data were anonymised, and all data processing was conducted in line with GDPR. Statistical analysis was undertaken using Microsoft Excel. Thematic analysis was applied to the free-text box responses.

**Result:**

16.49% (64/388) responded to the survey. Responses were uniform across all stages of training. 97% of respondents attended BG; 72% attended both formats, 25% attended only face-to-face and 3% online only. 65% of respondents preferred face to face compared to 18% online, whilst 11% stated no preference.

On thematic analysis, trainees asserted a preference for face-to-face, describing better group cohesion, feeling safer to share, increased ease of interpreting non-verbal communication, and that conversation was more fluid. They described greater ease of engagement with the group/facilitator and preferred direct social interaction with peers.

Conversely, most trainees acknowledged that online groups were convenient to attend, less time consuming & mitigated COVID risk associated with face-to-face meetings. Common themes against the use of online groups were: less psychotherapeutic in nature, technical issues, silences, unable to see participants faces and as though speaking “into the void”.

Regarding trainees' attitudes to attending BG, most of the trainees found BG had been beneficial in developing more interest and confidence in dealing with the psychological aspects of patient care. Trainees agreed that skills improved in developing an excellent doctor-patient relationship, recognising patients under stress/ in distress, systemically obtaining psychological information and making treatment decisions based upon psychological needs and psychotherapeutic engagement. They agreed that they could better understand the influence of doctors' emotions on the doctor-patient relationship.

**Conclusion:**

This survey showed that most trainees find BG beneficial in developing better doctor-patient relationships, preferring face-to-face rather than online BG. However, they found online more convenient. A blended learning approach could provide trainees with the benefits of both formats of BG.

